# Pollinator Behavior Drives Sexual Specializations in the Hermaphrodite Flowers of a Heterodichogamous Tree

**DOI:** 10.3389/fpls.2019.01315

**Published:** 2019-10-18

**Authors:** Eric Wajnberg, Noemi Tel-Zur, Idan Shapira, Yochai Lebber, Simcha Lev-Yadun, Udi Zurgil, Orna Reisman-Berman, Tamar Keasar

**Affiliations:** ^1^INRA Sophia Antipolis and: INRIA, Sophia Antipolis, Projet Hephaistos, France; ^2^French Associates Institutes for Agriculture and Biotechnology of Drylands, J. Blaustein Institutes for Desert Research, Ben-Gurion University of the Negev, Sde-Boqer, Israel; ^3^Department of Biology and Environment, Faculty of Natural Sciences, University of Haifa–Oranim, Tivon, Israel; ^4^Department of Natural and Life Sciences, Open University of Israel, Ra’anana, Israel

**Keywords:** dioecy, heterodichogamy, insect pollination, probabilistic model, Ziziphus spina-christi

## Abstract

Dioecy, the specialization of individuals into either male-only or female-only sexual function, has multiple evolutionary origins in plants. One proposed ancestral mating system is heterodichogamy, two morphs of cross-fertilizing hermaphrodite flowers that differ in their timing of flowering. Previous research suggested that small specializations in these morphs’ functional genders could facilitate their evolution into separate sexes. We tested the possible role of pollinators in driving such specializations. *Ziziphus spina-christi* is an insect-pollinated heterodichogamous tree with self-incompatible flowers and two sympatric flowering morphs. We compared the flower development patterns, floral food rewards, pollinator visits, and fruit production between the two morphs. Male-phase flowers of *Z. spina-christi*’s “Early” and “Late” morphs open before dawn and around noon, respectively, and transition into female-phase 7–8 h later. Flowers of both morphs contain similar nectar and pollen rewards, and receive visits by flies (their ancestral pollinators) at similar rates, mostly during the morning. Consequently, the Early morph functions largely as pollen donor. The Late morph, functioning as female in the morning, produces more fruit. We developed an evolutionary probabilistic model, inspired by *Z. spina-christi*’s reproductive system, to test whether pollinator visit patterns could potentially play a role in an evolutionary transition from heterodichogamy towards dioecy. The model predicts that reproductive incompatibility within flowering morphs promotes their evolution into different sexes. Furthermore, the pollinators’ morning activity drives the Early and Late morphs’ specialization into male and female functions, respectively. Thus, while not required for transitioning from heterodichogamy to dioecy, pollinator-mediated selection is expected to influence which sexual specialization evolves in each of the flowering morphs.

## Introduction

Dioecy entails the division of sexual function and reproductive effort between male-only and female-only individuals. This mating system occurs in only approximately 5% of land plant species, but exists in many different phylogenetic clades (Renner, 2014; Henry et al., 2018). It is widely agreed that dioecy in Angiosperms has repeatedly evolved from an ancestral hermaphroditic mating system (i.e., flowers with both male and female function), probably through several alternative pathways. These include gynodioecy (female and hermaphrodite plant individuals), androdioecy (male and hermaphrodite plant individuals), or monoecy (male and female flowers within each plant individual) as transitional states (Delph and Wolf, 2005; Henry et al., 2018).

Several hermaphroditic mating systems include two genetically determined morphs of individuals that are cross-compatible, thus individuals of different morphs can interbreed. The two morphs sometimes also differ in the style’s length (distyly, a special case of heterostyly — a polymorphism in style length), in the style’s orientation (enantiostyly), or in the timing of flowering (heterodichogamy, also termed synchronous dichogamy). Such dimorphism was proposed to lead to the evolution of dioecy (see Duan et al., 2018, for a recent discussion). The first step in such a possible evolutionary transition is probably a genetic mutation that disrupts either the male or the female sexual function in one of the morphs. Hence, that morph becomes unisexual while the other morph remains hermaphrodite, leading to either androdioecy or gynodioecy. Later mutations that disable the opposite sex in the hermaphrodite morph then become selectively advantageous, culminating in dioecy (Delph and Wolf, 2005). Everything else being equal, both morphs are as likely to evolve into male as into female plants. There are situations, however, where the morphs are slightly specialized toward a male or a female functional gender. The morphs can differ in traits such as resource allocation to vegetative growth *vs.* reproduction, the timing of growth, and floral nectar production (Atsatt and Rundel, 1982; Delph, 1990). Such an initial bias is predicted to increase during the evolution towards dioecy. A morph that produces more offspring through pollen export (male reproductive function), than through pollen import (female reproductive function), in a distylous or heterodichogamous species, is predicted to evolve into a pure male morph under dioecy. Conversely, a morph that achieves more of its mating success through the female reproductive function (bearing fruit) than through its male function (exporting pollen), will evolve into pure female (Pannell and Verdú, 2006).

Heterodichogamy, the focus of the present study, occurs in various species in three variants, all involving two different flower morphs. The first variant comprises one morph that switches from producing male-only flowers to producing female-only flowers along the blooming season, and a second morph that behaves in the opposite way, i.e., first produces female flowers, and only later in the season the male flowers. In the second variant, all plants produce hermaphroditic dichogamous flowers, but in one of the morphs the flowers are protandrous (male-first), while in the second morph they are protogynous (female-first). Plants of the third variant produce flowers that are either all protandrous or all protogynous, but open at different times of the day. Consequently, in all variants, male-phase flowers of one morph coincide with the female-phase flowers of the other morph (Renner, 2001). In a few heterodichogamous species, one of the morphs acts mainly as the pollen donor while the other morph functions primarily the as pollen recipient. These partial sexual specializations were suggested to facilitate the evolution of dioecy (Dommee et al., 1990; Pendleton et al., 2000; Pannell and Verdú, 2006; Gleiser et al., 2008).

Pollinators are important agents of selection on diverse plant traits, including flowering phenology, visual and chemical floral signals and inflorescence structure (Willmer, 2011). Beach (1981) hypothesized that pollinator-mediated selection can also promote the evolution of dioecy. Foraging pollinators prefer visiting highly attractive plants (such as plants with large inflorescences or those offering high pollen and nectar rewards) over less attractive individuals. The male reproductive success of plants increases linearly with pollinator visit rate while female reproductive success quickly saturates, hence components of males’ reproductive success depend more strongly on insect visit rates than the female reproductive success components (Charnov, 1979). Therefore, highly attractive and rewarding genotypes will outperform less attractive or rewarding ones in male function but not necessarily in the female function. This initial asymmetry can hypothetically promote the specialization of the more attractive individuals into pure males, and of the less attractive individuals into pure females, which can in turn push the reproductive system to evolve to dioecy.

Here we considered a similar hypothesis regarding the heterodichogamous *Ziziphus spina-christi* (Rhamnaceae), in which flowers of the two morphs open at different times during the day. We asked whether insect pollinators potentially can play an important role in the plant’s gender divergence and specialization. To identify possible gender asymmetries between the two flowering morphs of *Z. spina-christi*, we tested whether they differ in their flower development pattern, floral food rewards, pollinator visits, and fruit production. We then developed a simple probabilistic model, inspired by our case study, to test whether pollinator visit patterns could potentially play a role in an evolutionary transition towards dioecy. Models for the evolution of dioecy have traditionally stressed its benefits in avoiding self-fertilization, for plants that lack physiological self-incompatibility (de Jong and Klinkhamer, 2005; but see Routley et al., 2004). Our hypothesis, on the other hand, is not limited to self-compatible species but also applies to self-incompatible plants.

## Materials and Methods

### Study Organism

*Z. spina-christi* is a local arid-zone tree of African origin (Galil and Zeroni, 1967). The insect-pollinated trees produce large numbers of hermaphrodite flowers. As in many other heterodichogamous plants (Sun and Verdú, 2016), the flowers are short-lived (up to 48 h). Flowers are small, radially symmetrical, and lack a corolla tube. They are yellowish in color, secrete nectar and emit a weak scent. These floral traits conform to a fly-pollination syndrome, although species of the genus *Ziziphus* are visited by a wide range of insects, including honeybees, wasps and ants (Zietsman, 1990; Mishra et al., 2004). Thus, we consider flies as the main ancestral pollinators of *Z. spina-christi*.

**Figure 1:**
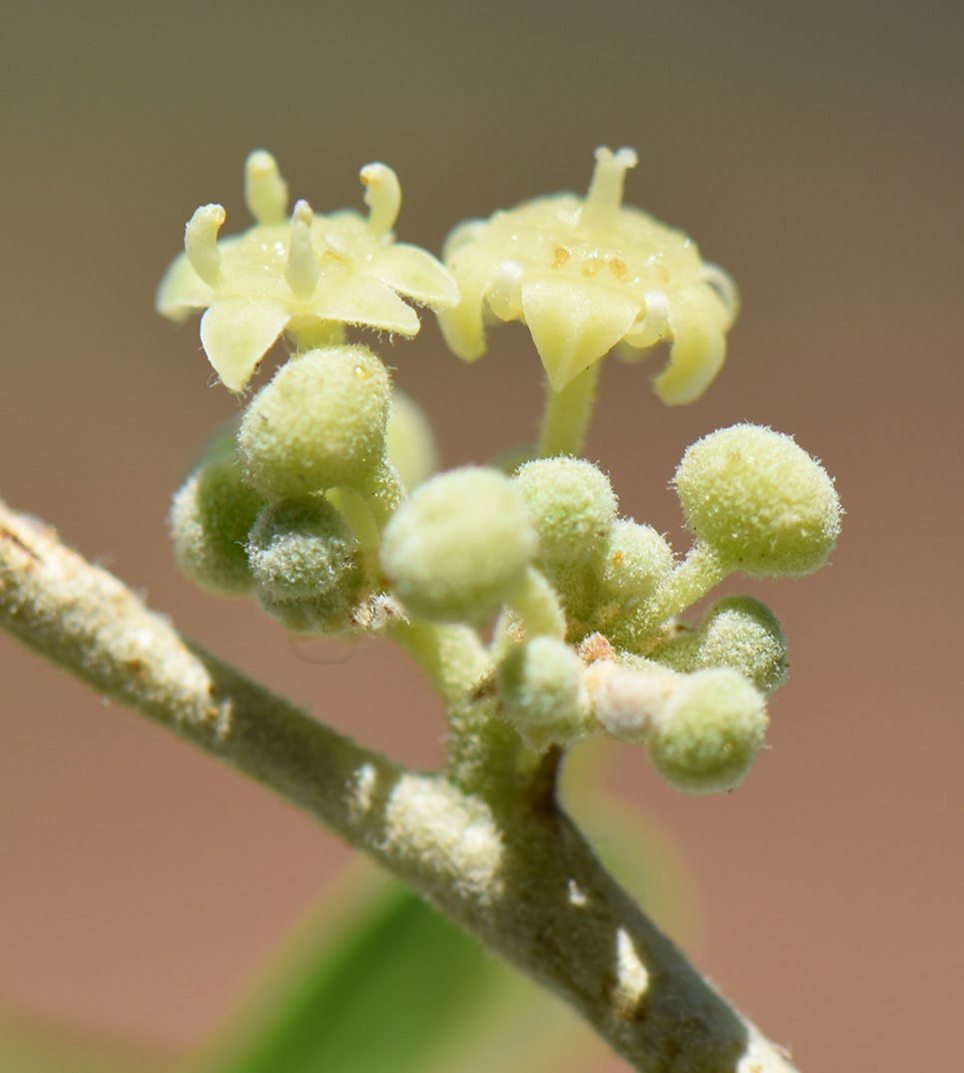
Male-phase (left) and female-phase (right) flowers of *Ziziphus spina-christi*. Flower diameter is 4–5 mm. Photo: S. Lev-Yadun.

*Z. spina-christi*’s heterodichogamous mating system involves two sympatric tree morphs, both of which produce protandrous flowers. Flowers of “Early” (also sometimes called “Type A”) trees open as males before dawn, become fully open and functional in the early morning hours and switch into the female phase by noon of their first day (**Figure 1**). The transition involves elongation of the styles and folding back of the petals, sepals, and anthers. Flowers of “Late” (“Type B”) trees open approximately at noon as males and switch to their female phase by sunset (Galil and Zeroni, 1967). Thus, Early flowers in a population reach their female phase while recently-opened Late flowers are still in their male phase. In the morning, after the Late flowers had attained their female phase, new fresh male-phase flowers of the Early trees are again available. This system allows cross-pollination between Early and Late trees. The synchronized floral development reduces encounters between male and female gametes of the same morph. It is unknown whether different individuals of the same morph are genetically cross-compatible. The species is self-incompatible, i.e., flowers on the same tree individual cannot cross-fertilize (Galil and Zeroni, 1967; Asatryan and Tel-Zur, 2013). This provides an additional mechanism that increases outbreeding. Binucleate pollen grains and the cessation of pollen tube growth in the style, rather than already on the stigma, suggest that the self-incompatibility system of *Ziziphus* is controlled at the gametophytic level (Asatryan and Tel-Zur, 2013). S-locus genes were proposed to control self-incompatibility at the gametophytic level in *Ziziphus jujuba* (Huang et al., 2016), but these findings could not be verified in a later molecular study in *Z. spina-christi* (Tel-Zur, unpublished results).

### Study Sites

*Z. spina-christi* trees were followed in two natural populations in Israel during the 2016–2018 flowering seasons. One population, Tabor Stream, is located in the north of the country (32°38′19″N, 35°26′50″E, elevation 90 m). Mean monthly temperatures in the area range between 11.4–27.9°C and the mean annual rainfall is 466 mm. The second population, Shoket, is located in a Mediterranean-desert transition zone in the south of the country (31°30′79″N, 34°90′16″E, elevation 350 m). It is characterized by an average yearly temperature range of 12.4–28.1°C, and an average annual rainfall of 213 mm. Observations on the timing of the flowering stages were also conducted in a planted and irrigated *Z. spina-christi* grove, composed of multiple genetic sources, at an experimental desert site in Sde-Boqer, southern Israel (30°85′34″N, 34°78′33″E, elevation: 480 m, temperature range: 9.9–26.2°C, average annual rainfall: 93 mm). Observations dates were chosen according to the availability of flowers in the three populations, and observation hours were adjusted to day length and insect activity.

### Monitoring of Flowering Morphs and Flowering Phenology

Sixteen trees from the Shoket population were monitored between 8:00 and 9:30 am, once a month during the 2016–2018 flowering seasons. We recorded whether the trees were in bloom, and whether blooming trees carried male-phase flowers. We used this information to determine the period of peak blooming in this population, and to assess whether the trees displayed a consistent flowering morph along and between seasons.

### Monitoring of Flower Development

Flower development was followed in the Shoket population in July and September of 2018 and in the Sde-Boqer grove in October of 2018. We marked several branches of Early (n = 3 in Shoket, n = 2 in Sde-Boqer) and Late (n = 3 in Shoket, n = 3 in Sde-Boqer) trees, which carried many large buds, and removed all open flowers. The same tree individuals were marked in Shoket on both dates. The number of marked branches was adjusted to attain at least 50 flower buds per tree that would open on the next day. Starting on the next morning (for Early trees) or early afternoon (for Late trees), we monitored the trees during the main activity hours of pollinators (7:30–19:00 in July, 7:30–17:00 in September, 8:00–15:00 in October) during two consecutive days. Monitoring rounds were conducted at 7:30, 11:00, 13:00, 15:00, 17:00 (in July and September only), and 19:00 (in July only). Each flower was monitored up to nine times during its two days of flowering. We recorded the numbers of flowers in each developmental stage, as defined by Galil and Zeroni (1967).

In some flowers, the styles do not elongate and the stigmas remain undeveloped. Although Galil and Zeroni (1967) mentioned these flowers and suggested that they do not have a female function, they did not include them in their classification of flower phases. We recorded the numbers of flowers with undeveloped styles while following flower development. We also collected flowers with undeveloped styles from trees of the Early (n = 8 flowers) and Late (n = 7 flowers) morphs in the Sde-Boqer population. We used the aniline blue staining protocol (Mori et al., 2006) to determine the frequency of flowers with germinated pollen grains and grown pollen tubes, as a measure of the flowers’ potential female function. We compared these flowers with a control sample of female-phase flowers with well-developed styles (Early morph: n = 10, Late morph: n = 13 flowers).

### Floral Rewards

*Z. spina-christi*’s insect visitors feed on the protein-rich pollen and on the carbohydrate-rich nectar in the flowers. The number of pollen grains produced per flower was estimated in the Tabor Stream and Shoket populations in 2016 as a measure of pollen rewards. We collected five large flower buds from each of 26 trees (Tabor Stream: eight Early and eight Late trees; Shoket: four Early and six Late trees). The buds were placed on ice in the field and frozen (−20ºC) in the laboratory until further treatment. In the laboratory, we separated five anthers from each flower bud and estimated the number of pollen grains per anther as described by Tel-Zur and Schneider (2009).

Nectar standing crops were estimated from flowers that were accessible to insect visitors in July and September 2017 in the Tabor Stream population, and in July and September 2018 in the Shoket population. We collected flowers from each of five Early trees and five Late trees in the morning (8:00–10:00) and in the afternoon (14:00–16:00) of each sampling date. The flowers from the Early (respectively, Late) trees were in anthesis or male phase when collected in the morning (respectively, afternoon), and in a transition or female phase when collected in the afternoon (respectively, morning). The flowers were immediately placed on ice in the field, transported to the laboratory and kept frozen (−20ºC) until their nectar content was measured. We used a modification of the nectar washing technique (Adgaba et al., 2017) for nectar measurements. The flowers were defrosted at room temperature, which ranged 17–26ºC. The crystallized sugar on the floral receptacle was dissolved in 5 μl distilled water for 2.5 min. The water droplet was collected using a pipette and its sugar concentration was measured with a hand-held refractometer suitable for the 0–10% sugar concentration range. Most nectar standing crops were measured from ten randomly sampled flowers per tree per sampling session. Damage to some frozen flowers and measurement errors occasionally reduced the number of replicates per sample.

### Insect Visits

Insect visits were recorded on the same trees, dates and times of day on which flowers were collected for nectar sampling. Namely, in July and September 2017 in Tabor Stream, and in July and September 2018 in Shoket. *Z. spina-christi* flowers are arranged in small clusters (inflorescences) along the branches. There are on average 7.80 ± 0.41 (SE) flowers per cluster at peak bloom. We recorded the number of insect visits (i.e., landings) on a known number of flower clusters for 10 min in each tree. We distinguished between the following visitor groups: flies, honeybees, non-*Apis* bees, wasps, ants, butterflies, and other insect visitors. Although these groups of visitors vary in their effectiveness as pollinators, frequency of visits is a good proxy for pollinator importance at the community level (Ballantyne et al., 2017).

### Flower and Fruit Numbers

We collected five small (about 20 cm long) branches from each of the flowering trees in the Shoket population in July 2017 (12 trees) and in July 2018 (15 trees, 11 of them also sampled in 2017). The branches were collected during the morning hours (8:00–9:30 am). We counted the male-phase flowers, female-phase flowers and fruit at any developmental stage in each cluster along each small branch. This provided a snapshot in time of the flowering and fruiting success in the two morphs, because the clusters’ position along each small branch, from distal to proximal, correlates with their age.

### Data Analysis

#### Flower Development

We used a generalized linear mixed model for binomial data with a logit link function to test the effects of tree morph (Early *vs.* Late), time of day (morning *vs.* afternoon), monitoring round (1–9, a correlate of floral age class), and the interactions between tree morph and the two other factors, entered as fixed factors, on the proportion of male-phase flowers. We ran three additional similar models to evaluate the effects of the same explanatory variables on the proportions of transition-phase, female-phase and sterile female flowers. We used the number of flowers in each observation as a weighing covariate. In all models, sampling date (two dates in Shoket, one in Sde-Boqer) and tree ID were added as fixed-intercept random factors to account for the repeated sampling design.

#### Floral Rewards

The distribution of the estimated number of pollen grains per flower per tree conformed to normality assumptions. We used a Gaussian linear mixed model to test for the effects of flowering morph as a fixed factor, with population as a random factor, on the pollen loads. Floral sugar mass values from each sampling session were averaged per tree and log-transformed to meet normality assumptions. We then tested the effects of tree morph (Early *vs.* Late) and time of day (morning *vs.* afternoon) as fixed factors on the nectar standing crops. Tree ID and sampling date were included as random factors. In this case, we ran a separate model for each population (Tabor Stream and Shoket), because the time of day seemed to have opposite effects on nectar standing crops in these two populations.

#### Insect Visits

We divided the number of visits by the number of flower clusters in each 10-min observation period. We then used a Gaussian linear mixed model to test the effects of time of day and flowering morph as fixed factors on fly visit rates. Population (Tabor Stream or Shoket), sampling date and tree ID were included in the model as random factors.

#### Flower and Fruit Numbers

We averaged the numbers of flowers and the numbers of fruit per cluster, separately for each tree-sampling date combination, over the five replicate branches per tree. Thus, each sampled tree was treated as a unit of replication. We used linear mixed models to test the effects of cluster number and flowering morph as fixed factors, separately on the per-cluster flower numbers and on fruit numbers. Tree number and year were included as random factors in the models, to account for the dependence between branches within trees, and between trees that were sampled in both years.

All statistical analyses were done with R version 3.5.1 (R Core Team, 2018). The functions “glmer” and “lme” of the packages “lme4” and “nlme” and the package “lmerTest” were used for the linear mixed models (Bates et al., 2015; Kuznetsova et al., 2017).

### The Probabilistic Model

To explore whether dioecy can evolve from heterodichogamy, and whether such an evolutionary change can be mediated by pollinators, we developed a probabilistic framework. This approach complements previous analytical models of the evolution of plant breeding systems (de Jong and Klinkhamer, 2005) by explicitly incorporating the foraging patterns of pollinators. The framework is inspired by the flowering biology of *Z. spina-christi*, but does not intend to capture all its details. In it, a tree population was modeled as initially comprising 50 Early and 50 Late individuals that rely entirely on insect pollination. Among all prospective pollinators, we focused on the flies, because, according to flower structure, they are the presumed ancestral pollinators of *Ziziphus* (Zietsman, 1990; Mishra et al., 2004). Thus, they are potentially important agents of selection on flowering in the genus. In addition, flies were the most common flower visitors in our field observations (see Results section). The flowers of each morph bloom in perfect synchrony and are potentially completely self-incompatible. There are two times per day: morning and afternoon, in which all protagonists are present. During each half-day considered in the model, trees of each flowering morph receive a fraction of the overall number of pollinator visits, and these fractions add up to 1. In other words, the probability distribution of visits over the half-days for the two flowering morphs is computed from the average number of visits *V* *_ij_* in the *i* ^th^ half day (morning or afternoon) to the *j* ^th^ flowering morph (Early or Late) using the following equation:

(1) $$P_{ij}=\frac{v_{ij}}{\Sigma_{i}\Sigma_{j}v_{ij}}\;(\Sigma_{i}\Sigma_{j}P_{ij}=1)$$

The trees’ sex allocation in the model (as in several plant species; Sato, 2002) is assumed to be genetically determined. Several lines of evidence point to genetic control over the timing of flowering events. Such events include switching from vegetative growth to flowering (Stinchcombe et al., 2004; Buckler et al., 2009), and transitioning between the production of male and female flowers (Friedman and Barrett, 2011) or sexual phases (Routley and Husband, 2005). Similarly, in our model, we assume that a tree’s genotype controls its time of switching from male-phase to female-phase. In the beginning, and independently in the two flowering morphs, the genotypes of all individuals are drawn randomly, leading each individual to have a genotype *g* uniformly distributed in the [0; 1] interval. *g* defines the proportion of a flower’s lifetime spent in the male phase. Hence, an individual having genotype *g* = 1 will produce male flowers only, while an individual having genotype *g* = 0 will produce female flowers only.

**Figure 2:**
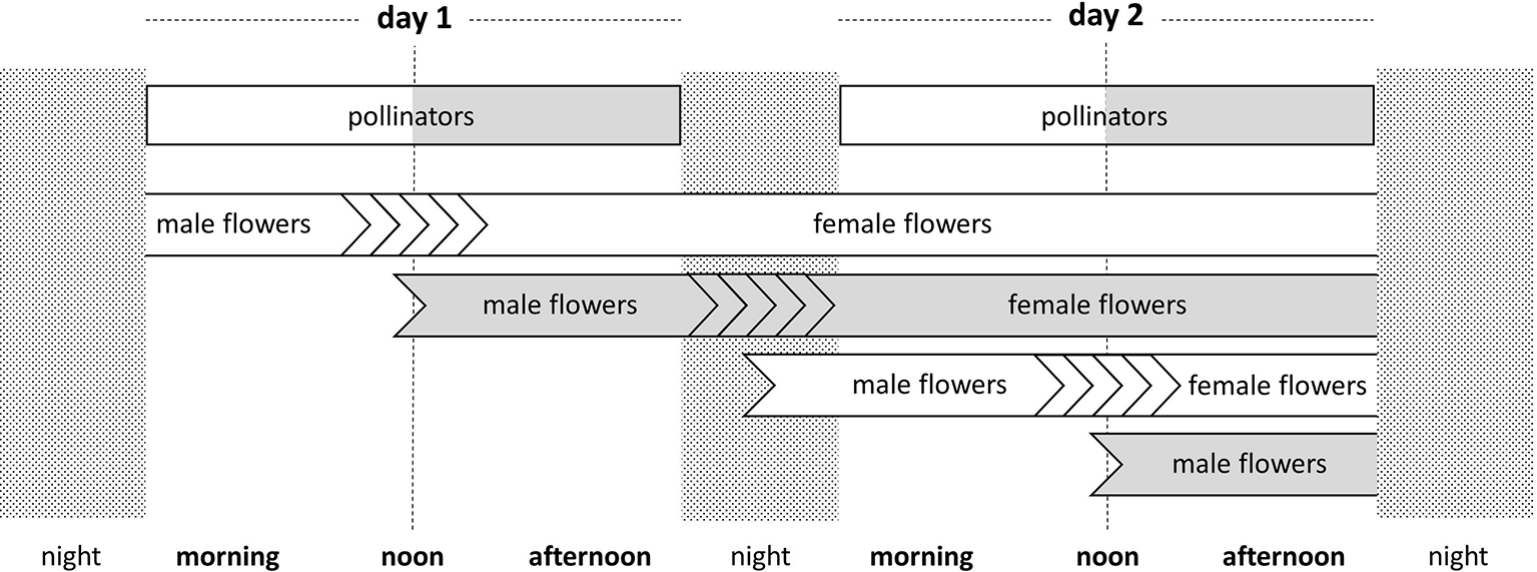
Time course of the flower sex phases in the two protandrous flowering morphs over two consecutive days, as used in the probabilistic model. The modeled time unit is 48 h, the blooming duration of a single flower. The timing of visits by morning-active and afternoon-active pollinators, as incorporated into the model, is indicated. Open bars represent the Early flowering morph and morning pollinators. Grey bars represent the Late flowering morph and afternoon pollinators. Multiple chevrons represent time variation intransitions from male- to female-phase. The events in this 48-hour time unit are representative of the whole flowering season.

Each flower blooms during two consecutive days (i.e., 48 h). In order to estimate the proportion of male *m* and female *f* flowers of a given genotype *g* in a given morning or afternoon period, the beginning and the end of the period were first transformed into fractions of the two consecutive days simulated. Then, a genotype *g* was considered to produce male (respectively, female) flowers only, if *g* is greater than the end of the period considered (respectively, lower than the beginning of the period). An individual having a genotype *g* between these two extreme values was considered to produce fractions of male and female flowers in proportion to the value of *g* within the beginning-end time interval. For example, *g* = 0.5 corresponds to switching from male to female 24 h after the opening of the flowers. Within the time interval of 20–28 h, this genotype functions equally as a pollen donor (during 20–24 h) and as a pollen recipient (during 24–28 h). The time schedule of reproductive events of the different flowers of the two flowering types is depicted in **Figure 2**. Early trees start their cycle at 4:00 am while Late trees start it at noon.

A tree’s potential to dispense (respectively, to receive) pollen was calculated as the proportion of time it spends in the male (respectively, female) phase in the four half-days (first and second mornings and first and second afternoons). The male component of the fitness of a tree genotype was considered to depend on the proportion of time it spends in the male phase, on the intensity of pollinator activity, and on the availability of compatible female-phase flowers during that time. Reciprocally, the female component of the fitness of a tree genotype is affected by the proportion of time spent as female, by pollinator activity and by the availability of suitable male-phase reproductive partners. Males must find females to reproduce and this happens with a frequency that can be estimated by the sum of all female frequencies over the entire population, *i.e.*, Σ*_genotypes_* *f*. These females need also to be visited by pollinators, with probability *P* *_ij_*. Hence, the contribution of males of a given genotype to reproduction, in the *i* ^th^ half day and for the *j* ^th^ flowering morph, can be estimated using the following equation:

(2) $$mP_{ij}\Sigma fP_{ij}$$

The same reasoning was used to compute the contribution of females of each genotype. We calculated fitness values for all of the eight, mutually independent situations (four half-day × two sexes) for each genotype and summed them up to produce each tree’s fitness. This fitness score combines the tree’s male and female fitness components. Finally, to take into account potential intra-genotype auto-incompatibility, we removed each tree’s genotype from the sum in Equation 2 above. Similarly, incompatibility between trees of the same flowering morph was considered by removing Early genotypes as potential reproductive partners when computing fitness for Early trees, and reciprocally for the Late trees.

At the end of the 48 h potential fertilization phase, the probability of each genotype to contribute to the next generation was estimated by dividing each fitness score by the sum of all scores in the population, and this was used to build a new population of 100 trees. In this process, progeny-producing trees passed on to their descendants both their flowering morph and their genotype. For this, the genotype of each tree in the next generation was drawn randomly from the pool of genotypes present in the previous generation, with a probability proportional to its relative fitness. Thus, the genetic mechanisms of genotype inheritance (e.g., dominance, mutation, and recombination) are not explicitly modeled. The process was repeated for 200 generations to predict evolutionary trajectories that describe changes in the distribution of tree genotypes.

To test whether the pollinators' activity pattern can select for dioecy in the floral system modeled here, we simulated the following visit rate distributions: (a) all visits occur in the morning; (b) all visits occur in the afternoon; (c) identical visit rates in the morning and in the afternoon; (d) the actual distribution of pollinator visits observed in the field. We considered separately the distribution of visits by flies (the presumed ancestral pollinators of *Ziziphus*), as well as the distribution of visits by flies and honeybees combined (the main flower visitors in the field observations). To examine whether self-incompatibility plays a role in the evolution of dioecy, we also ran simulations with the field-observed distribution of fly visits, while eliminating the incompatibility within a genotype, within a flowering morph, or both of them. The reproductive potential of flowers may decrease as they age, due to pollen depletion, declines in stigma receptivity, or both. To explore the sensitivity of the model’s predictions to such possible age-related declines in floral fertility, we repeated the calculations while introducing reduced contributions of day-2 flowers to the trees’ fitness. We simulated a 50% decrease in the contribution of day-2 flowers to the male fitness component, to the female fitness component, and to both the male and the female fitness components.

The model tests the hypothesis that trees playing a pure dioecy strategy (all individuals being either *g* = 0 or *g* = 1) have a fitness advantage and will increase their frequency in the population. According to this hypothesis, the distribution of *g* in the population is expected to shift gradually from uniform to bimodal. To further explore our hypothesis, we also constructed fitness plots for the different modeled scenarios by simulating a population with 101 Early and 101 Late trees, each having genotypes ranging from 0.0 to 1.0 by steps of 0.01 (i.e., 101 different genotypes corresponding to 101 individuals for each flowering type). In this case, we calculated the fitness of each genotype after a single generation only.

## Results

### Consistency of the Flowering Morphs and Flowering Phenology

Flowering in the Shoket population started in June, peaked in July and ended in December: Each tree consistently displayed the same flowering morph (Early: n = 8 trees, Late: n = 8 trees) throughout the three years of monitoring.

### Flower Development

**Figure 3:**
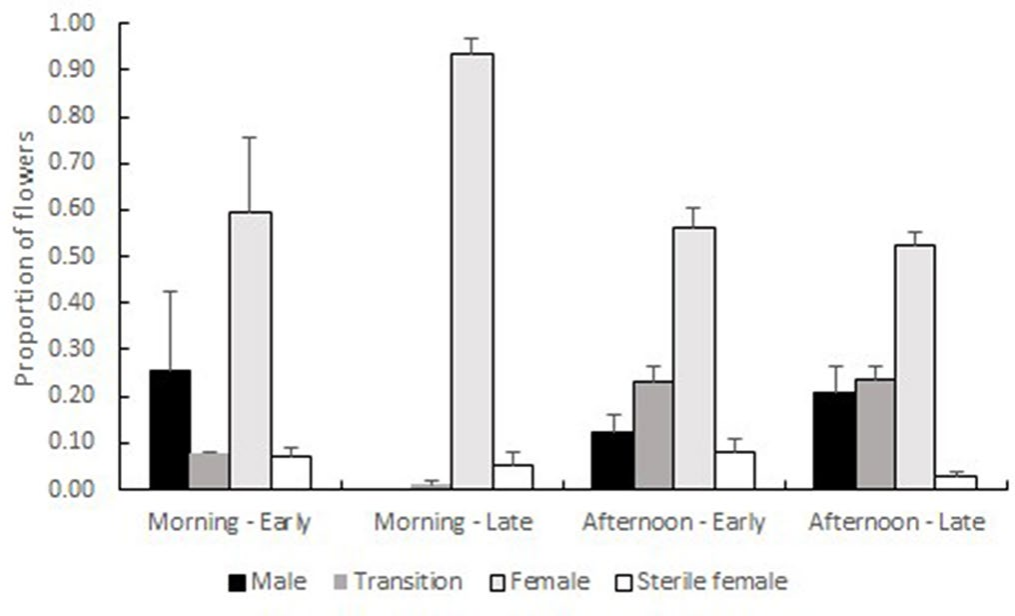
Proportions (+ SE) of male-phase, transition, female-phase and female-sterile flowers on Early (n = 5) and Late (n = 6) trees, sampled in the morning and in the afternoon. Proportions are averaged over the three samples (July and September 2018 in Shoket, October 2018 in Sde-Boqer). All viable-looking flowers were counted, reflecting the flowers that had opened on the day of counting and on the day that preceded it. Time of day, but not flowering type, significantly affected the proportions of transition- and female-phase flowers. The proportions of male-phase and female-sterile flowers were not influenced by either main factor. The interaction between time of day and flowering type significantly affected the proportions of male-phase, transition- and female-phase flowers. See **Table 1** for a full summary of the statistical tests.

Early trees in both the Shoket (n = 3) and in the Sde-Boqer (n = 2) populations carried higher proportions of male-phase flowers, and lower proportions of female-phase flowers in the mornings, as compared with Late trees of the same populations (n = 3 trees in each population). The opposite pattern occurred in the afternoons (**Figure 3**). **Table 1** summarizes the statistical hypotheses tested to explain these patterns. Time of day as main effect, but not flowering morph, significantly (*P* < 0.05) affected the proportions of transition- and female-phase flowers. The proportions of male-, transition- and female-phase flowers were significantly influenced by the interaction between the time of day and flowering morph.

**Table 1:** Summary of generalized linear mixed models, testing for factors influencing the proportions of male, transition, female, and sterile female flowers. The effects of the main factors in the models are reported, as well as the two-way interactions that involved flowering morph. Statistically significant effects (p < 0.05) are indicated in bold. Five Early trees and six Late trees were monitored.

	Fixed factors				
Response variable	Time of day	Flowering morph	Monitoring round	Morph × Time of day	Morph × Monitoring round
Degrees of freedom	1	1	8	1	7
Proportion of male-phase flowers	χ^2^ = 0.35 *P = 0.552*	χ^2^ = 0.09 *P* = 0.766	**χ** **^2^** ** = 4283.20** ***P*** ** < 0.001**	**χ** **^2^** ** = 8.70** **P = 0.003**	**χ** **^2^** ** = 339.44** ***P*** ** < 0.001**
Proportion of transition-phase flowers	**χ** **^2^** ** = 174.84** ***P*** ** < 0.001**	χ^2^ = 0.90 *P* = 0.343	**χ** **^2^** ** = 2956.30** ***P*** ** < 0.001**	**χ** **^2^** ** = 275.47** ***P*** ** < 0.001**	**χ** **^2^** ** = 1067.10** ***P*** ** < 0.001**
Proportion of female-phase flowers	χ**^2^** ** = 36.83** ***P*** ** < 0.001**	χ^2^ = 0.00 *P* = 0.966	**χ** **^2^** ** = 5108.60** ***P*** ** < 0.001**	**χ** **^2^** ** = 39.00** ***P*** ** < 0.001**	**χ** **^2^** ** = 189.23** ***P*** ** < 0.001**
Proportion of sterile female flowers	χ^2^ = 2.62 *P* = 0.105	χ^2^ = 0.80 *P* = 0.372	**χ** **^2^** ** = 320.15** ***P*** ** < 0.001**	χ^2^ = 3.06 *P* = 0.08	**χ** **^2^** ** = 44.67** ***P*** ** < 0.001**

None of the female-sterile flowers carried germinated pollen grains on the stigmas. On the other hand, 91% of the stigmas of female-phase flowers, carried at least one germinated pollen grain. These results confirm the loss of the female function in flowers with undeveloped styles.

### Floral Rewards

**Figures 4:**
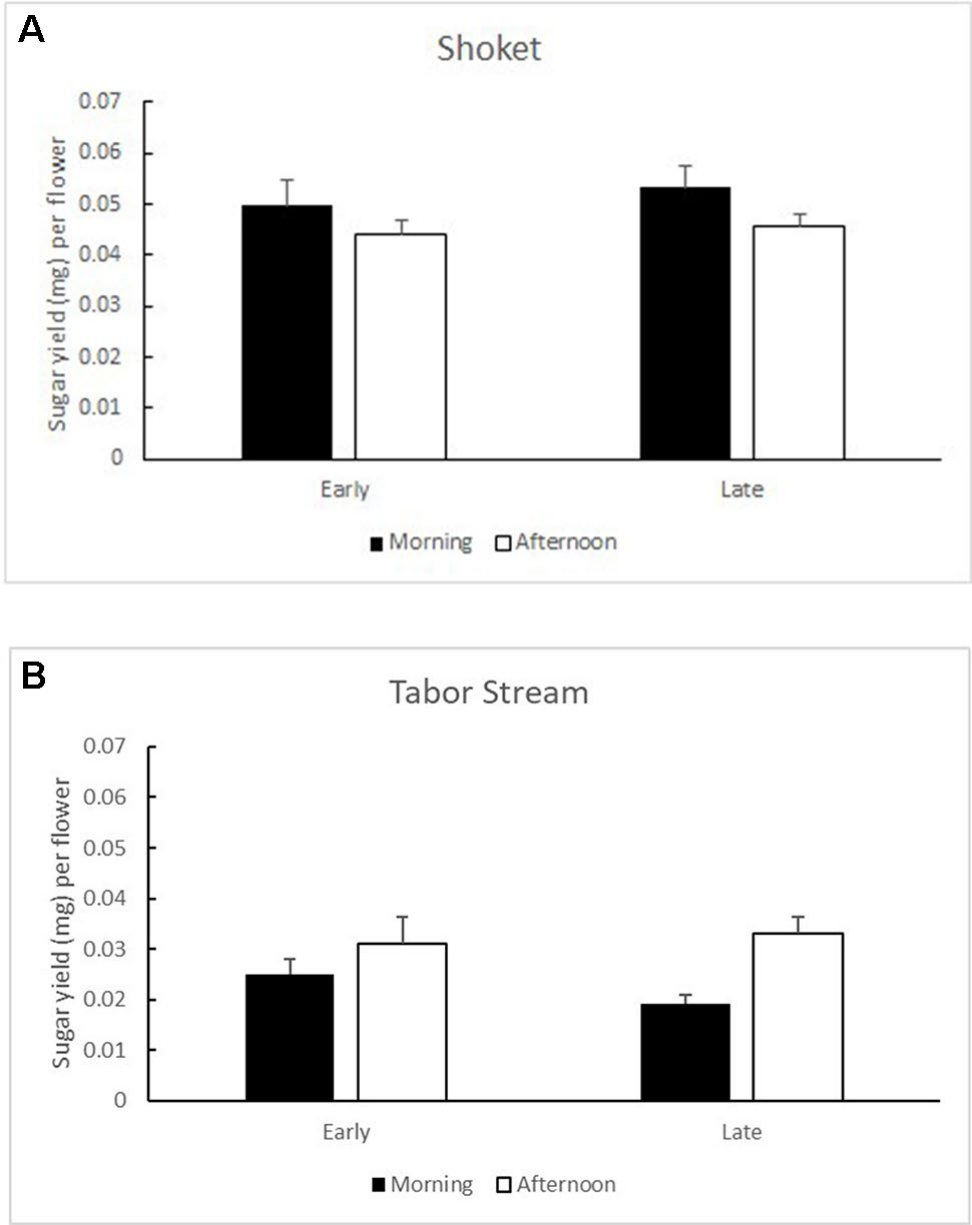
Mean (+ SE) sugar yield per flower in Early (left) and Late (right) trees, sampled in the morning (black bars) and in the afternoon (white bars) in the two study populations: **(A)** Shoket and **(B)** Tabor Stream.

Mature flower buds contained 3,798.73 ± 387.02 (SE) pollen grains per flower as potential food rewards. Flowering morph did not significantly affect the pollen loads (F_1,23_ = 0.095, P = 0.76). Nectar standing crops in the Shoket population were not influenced by the flowering morph (F_1,14_ = 0.215, P = 0.65) or by the time of day (F_1,18_ = 3.484, P = 0.08; **Figure 4**). In the Tabor Stream population, the time of day significantly affected nectar standing crops (F_1,13_ = 18.416, P = 0.001), but flowering morph did not (F_1,18_ = 0.322, P = 0.58; **Figures 4A and B**).

### Insect Visits

**Figure 5:**
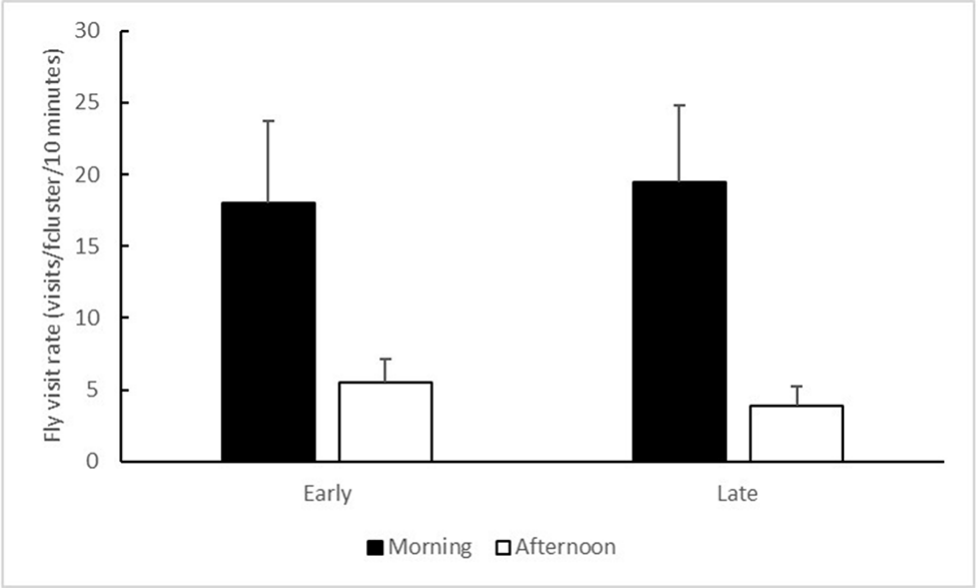
Mean (+ SE) numbers of fly visits per flower cluster over a 10-min observation period, conducted in the morning (black bars) and in the afternoon (white bars). Data from the Tabor Stream and the Shoket populations were combined, because 78.9% of all visits occurred at the Tabor Stream site.

The flowers were visited by a variety of insects, including flies (40.2% of the visits, dominated by the families Muscidae and Calliphoridae), honeybees (34.4% of the visits, mostly at the Tabor Stream site), ants (8.0%), wasps (7.0%), small bees (6.0%), butterflies (2.5%), bugs (0.8%), and others (1.1%). 78.9% of all observed visits occurred in the Tabor Stream population. The frequencies of fly visits were higher in the morning than in the afternoon (time of day: F_1,69_ = 22.753, *P* < 0.001; **Figure 5**). They were unaffected by the flowering morph (F_1,69_ = 0.025, *P* = 0.88) or by the time of day × morph interaction (F_1,69_ = 0.176, *P* = 0.68).

### Flower and Fruit Counts

**Figure 6:**
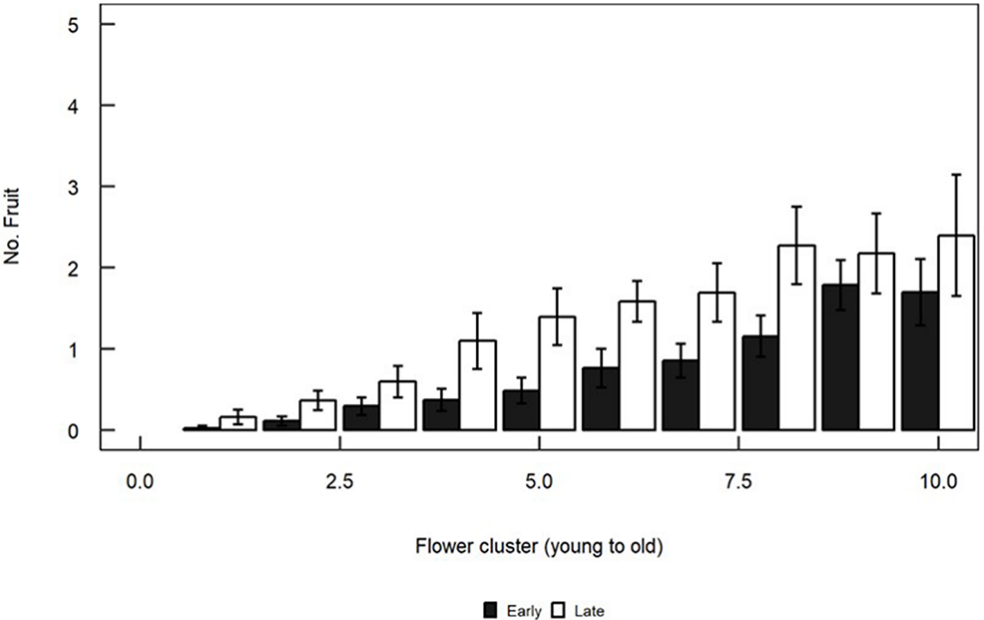
Mean (+/- SE) numbers of fruit per flower cluster in Early (black) and Late (white) trees. Clusters are numbered from distal (youngest) to proximal (oldest). Fruits were counted in five branches of each of the flowering trees in the Shoket population. Only means for clusters which are based on counts of >50 branches (clusters 1–10) are plotted.

The numbers of flowers varied significantly with cluster (F_13,216_ = 3.167, P < 0.001), but were not affected by the flowering morph (F_1,24_ = 2.441, P = 0.13) or by the morph × cluster interaction (F_13,216_ = 0.539, P = 0.90). Most fruit were found in the proximal (older) clusters. Late trees produced consistently more fruit than Early trees (**Figure 6**). Flowering morph (F_1,24_ = 5.946, P = 0.02), cluster number (F_13,216_ = 22.917, P < 0.001) and the interaction between these two main factors (F_13,216_ = 3.329, P < 0.001) all significantly influenced fruit numbers.

### Probabilistic Model

**Figure 7:**
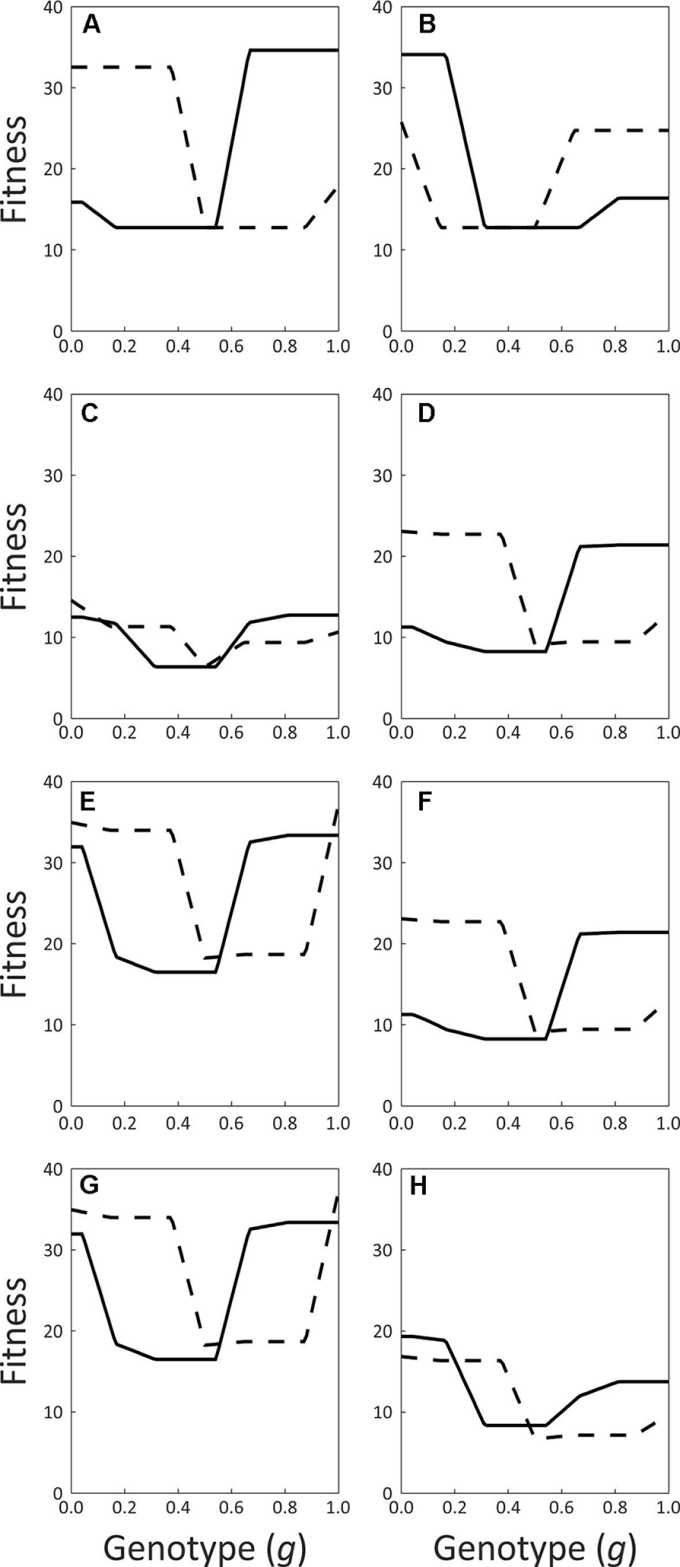
Functions describing the fitness (in arbitrary units) for all possible genotypes of the Early (solid lines) and Late (dashed lines) flowering morphs. **(A)** pollinators visit only in the morning, **(B)** pollinators visit only in the afternoon, **(C)** pollinators visit throughout the day, d-g: visits by flies, rates are as observed in the field. Mating incompatibility occurs within flowering morphs and genotypes **(D)**, within flowering morphs only **(E)**, within genotypes only **(F)** or never **(G)**. h: visits by honeybees and flies, rates are as observed in the field. See **Table 2** for additional details.

The simulations’ outcomes are summarized in **Table 2**, and the functions describing the fitness for all possible genotypes for Early and Late trees under the different simulated scenarios are plotted in **Figure 7**.Regardless of the daily pattern of pollinator visits, all of the simulations converged to subdioecy (incomplete unisexuality). 

**Table 2:** Summary of the results of the probabilistic simulation model done following eight different scenarios.

Scenario #	Distribution of insect visits	Within genotype Incompatibility	Within flowering morph incompatibility	Mean (± SD) of the final value of g		Mean (± SD) frequency of males evolved from		Fitness plot
				Early	Late	Early	Late	
1	20, 20, 0, 0 (morning only)	Yes	Yes	0.841 ± 0.098	0.183 ± 0.109	1.000 ± 0.000	0.000 ± 0.000	**Figure 7A**
2	0, 0, 20, 20 (afternoon only)	Yes	Yes	0.085 ± 0.049	0.821 ± 0.097	0.000 ± 0.000	1.000 ± 0.000	**Figure 7B**
3	20, 20, 20, 20 (throughout day)	Yes	Yes	0.821 ± 0.265	0.142 ± 0.271	0.900 ± 0.302	0.100 ± 0.302	**Figure 7C**
4	18.0, 19.4, 5.5, 3.9 (visits by flies, as in the field)	Yes	Yes	0.852 ± 0.083	0.163 ± 0.114	1.000 ± 0.000	0.000 ± 0.000	**Figure 7D**
5	18.0, 19.4, 5.5, 3.9 (visits by flies, as in the field)	Yes	No	0.862 ± 0.101	0.432 ± 0.240	1.000 ± 0.000*	0.329 ± 0.265	**Figure 7E**
6	18.0, 19.4, 5.5, 3.9 (visits by flies, as in the field)	No	Yes	0.867 ± 0.076	0.170 ± 0.112	1.000 ± 0.000	0.000 ± 0.000	**Figure 7F**
7	18.0, 19.4, 5.5, 3.9 (visits by flies, as in the field)	No	No	0.839 ± 0.082	0.417 ± 0.241	1.000 ± 0.000*	0.318 ± 0.278	**Figure 7G**
8	22.7, 38.9, 39.0, 10.9 (visits by honeybees and flies, as in the field)	Yes	Yes	0.812 ± 0.279	0.236 ± 0.283	0.890 ± 0.031	0.110 ± 0.031	**Figure 7H**

The distribution of insect visits denotes the numbers of visits/cluster/10 minutes to Early and Late trees in the morning, and to Early and Late trees in the afternoon. Within-genotype and within-morph fertilizations are either excluded or allowed. The simulations predict the mean values of genotypes g, that defines the proportion of a flower’s lifetime spent in the male phase, evolved in the Early and Late morphs. They also predict the proportions of simulations where Early and Late morphs take over the male function after 200 generations. Results are based on 100 replicate simulations for each set of parameters.

*The Early flowering morph went extinct in a sizable proportion of the simulations (58% of cases in scenario 5, 57% of cases in scenario 7). In these cases, some of the Late trees evolved a male-biased genotype (g > 0.8) and the remaining Late trees evolved a female-biased genotype (g < 0.2).

That is, some of the trees in the population evolved to low values of *g* (<0.2) and functioned mainly as females, while the remaining trees functioned mainly as males, with *g* values of above 0.8. A single male-biased and a single female-biased genotype evolved in most simulations.

It clearly appears that the insects’ visit patterns did determine which morph specialized into each of the sexual functions. When pollinator visits occur only or mostly in the morning, the Early trees always evolve into a mostly-male function and the Late trees become mostly-female (**Table 2**, scenarios 1 and 4). On the other hand, when visits occur in the afternoon only, the opposite pattern is observed, with Late trees assuming the male function and Early trees taking over the female function (**Table 2**, scenario 2). Finally, when we simulated insect visits throughout the day, Early trees evolved into mainly-males in 90% of the replicates, and Late trees filled the mostly-male function in the remaining 10% of replicates (**Table 2**, scenario 3). The visit pattern observed in the field for flies and honeybees combined leads to a similar outcome, as these insects visited the flowers approximately all over the day (**Table 2**, scenario 8). We conclude that our model always eventually converges to subdioecy, and that pollinator behavior appears to represent an important selective force on the sexual role played by the two flowering morphs as subdioecy evolves. Inspection of the curves describing the fitness of the different possible genotypes in the population confirms that, when pollinators forage predominantly in the morning, Early trees gain their highest fitness at high *g* values (i.e., mostly-male function), while Late trees maximize their fitness at low *g* values (mostly-female function, **Figures 7A and D**). These functions are rather flat for a range of low *g* values for Late trees, and for a range of high *g* values for Early trees. In other words, Early trees have similar fitness whether they are mostly-male or fully-male, and Late trees are similarly fit whether mostly-female or fully-female. This agrees with the observed evolution of subdioecy rather than full dioecy in the simulations. When insects forage throughout the day, Early trees attain similar fitness with both high *g* values and low *g* values (**Figure 7C and H**). This can explain why they evolve into males in some replicate runs and into females in others.

Introducing reproductive compatibility within each flowering morph often leads to the elimination of the Early morph (**Table 2**, scenarios 5 and 7). Nevertheless, subdioecy evolved as some of the Late trees functioned as mostly-male and others functioned as mostly-female. Late trees had higher fitness than Early trees in these simulations, both for low and for high *g* values (**Figure 7E and G**). Allowing within-genotype compatibility, while preventing within-morph reproduction, did not affect the simulations’ outcomes (**Table 2**, scenario 4 *vs.* 6, **Figure 7D and F**). Reducing the contribution of day-2 flowers to the male, female, or both the male and the female fitness components, did not alter the model’s qualitative predictions in the eight scenarios (data not shown).

## Discussion

Our field observations support the possible role of flies, the putatively ancestral pollinators of the heterodichogamous *Z. spina-christi* (Zietsman, 1990; Mishra et al., 2004), in promoting functional gender differentiation between the two flowering morphs. Both morphs contained similar amounts of nectar and pollen rewards and were visited by flies at similar rates. Nevertheless, some asymmetry between the two morphs is possible because most fly visits occurred in the morning hours at our study sites. Consequently, Early trees probably functioned mostly as pollen donors whereas Late trees were largely pollen recipients. The afternoon pollen transfer, from the Late to the Early trees, likely occurred at a lower rate. The heterodichogamous *Z. mauritiana*, which was studied in India, also received most of its insect visits in the mornings, with peak activity around 10:00 am. This activity pattern was attributed to thermal constraints on the pollinators. In particular, fly activity declined sharply at temperatures above 35 ºC (Mishra et al., 2004). Sensitivity to high temperatures may also account for the flies’ peak activity in the morning in the present study, since it was conducted in two sites with a hot climate.

Early trees had more male-phase flowers in the morning hours, while Late trees had more female-phase flowers and set more fruit than Early trees. This probably reflects the female function of the Late flowers on their second day. The higher fruit-set of Late trees may indicate their incipient specialization into the female function. This possibility is in line with earlier reports of sexual specialization in heterodichogamous species of the genera *Acer*, *Grayia*, and *Thymelaea* (Dommee et al., 1990; Pendleton et al., 2000; Gleiser et al., 2008). The heterodichogamous species within the genus *Acer* produce flowers of one sex at the beginning of the blooming season, and switch to producing different flowers, of the other sex, later in the season. Fruiting success in *Acer opalus* declines towards the end of the season due to resource limitation (Gleiser et al., 2008). This reduces the female component of reproductive success in the protandrous morph and the male success in the protogynous morph. The protandrous morph is thus likely to gain higher fitness from the male function at the beginning of the season than from the female function at the end. This morph is therefore predicted to evolve into pure male, while the protogynous morph is expected to evolve into pure female in this species (Pannell and Verdú, 2006). Sexual specialization of the flowering morphs was also documented in the congeneric species *A. mono*, in which protogynous trees have higher fruiting success than the protandrous ones (Shibata et al., 2009). Morph-related sexual specializations have been described in the heterodichogamous shrubs *Grayia brandegei* (Amarantaceae), and *Thymelaea hirsuta* (Thymelaeaceae) as well. In both species a protandrous and a protogynous flowering morph coexist. In *G. brandegei*, the female function is impaired in the protandrous morph. This results in fewer female-phase flowers, fruits and seeds than in the protogynous morph (Pendleton et al., 2000). The opposite specialization occurs in *T. hirsuta*, namely higher fruit sets in the protandrous than in the protogynous flowers (Dommee et al., 1990).

We also observed lower numbers of fruits in distal inflorescences than in proximal ones along each small branch, in both flowering morphs. This trend is probably unaffected by sexual specialization and may reflect the younger age of the distal clusters, i.e., the shorter period available to them to mature flowers and fruit. Resource limitation experienced by the distal clusters (as they grow further away from the plant’s nutrient flow, or suffer from competition with the developing fruits in the proximal clusters) may also limit their investment in female reproductive function (Zhao et al., 2015).

The most common mating system within the Rhamnaceae is hermaphroditism combined with protandry and self-incompatibility (Routley et al., 2004). Heterodichogamy within the family is limited to some (Asatryan and Tel-Zur, 2013), but not all (Cerino et al., 2015) species of *Ziziphus*. Andromonoecy (the production of both bisexual and male flowers on the same plant individual) is reported in the genera *Alphitonia*, *Colubrina*, and *Trevoa*, but such a breeding system has not been proposed as an intermediate step in the evolution of dioecy. True dioecy is known from the genus *Rhamnus* (Midan and Schirarend, 2004). Thus, the pathway from hermaphroditism to dioecy in this family could involve heterodichogamy. More comparative research on the mating systems of plants within the Rhamnaceae is needed to address this issue.

Each tree in the Shoket population exhibited the same flowering morph consistently, both within and between flowering seasons. At a finer scale, however, we observed some temporal variation in the trees’ flowering schedules: the duration of the male phase increased, and the within- and between-tree synchrony in flowering stages decreased, towards the end of the flowering season (unpublished data). Similar plasticity in sex expression is known from several plant species, and has been suggested to influence the evolution and maintenance of dioecy (Delph and Wolf, 2005; Renner, 2014).

Our simulations always resulted in subdioecy, independent of the temporal pattern of insect visits. In most scenarios, one of the flowering morphs evolved to mostly-male function and the other evolved to mostly-female function (**Table 2**, scenarios 1–4, and 6). Visits that occur mainly in the morning (scenario 4), as observed in our field study for flies, are predicted to favor mainly-male function in Early trees, and mainly-female function in Late trees. When pollinator visits are scattered throughout the day (scenario 3 and scenario 8), as also observed in the congener *Z. mucronata* (Zietsman, 1990), dioecy is predicted to evolve as well, but without complete gender specialization of the two flowering morphs. In the remaining simulations (**Table 2**, scenarios 5 and 7), some of the Late genotypes took over the mostly-male function and other Late genotypes evolved into mostly-females. Thus, it seems that the evolution of separate sexes in the model is not driven by any specific pattern of pollinator activity. In scenarios 1–4 and 6 (**Table 2**), selection for dioecy may result from the incompatibility within the two flowering morphs. Under these scenarios, an individual can only reproduce with another individual that belongs to both the opposite sex and the opposite flowering morph. Hence, reproductive opportunities are maximized if all individuals of the same flowering morph also express the same sex. Accordingly, the fitness functions show that purely hermaphrodite genotypes (i.e., with *g* = 0.5) always have lower fitness than genotypes that specialize in being mostly male (high *g*-values) or mostly female (low *g*-values). Similarly, a game-theoretic model predicts that dioecy will evolve in hermaphrodite plants only if their costs of inbreeding are high (de Jong and Klinkhamer, 2005). Self-incompatibility within a flowering morph is established in most species with distyly (Barrett and Shore, 2008). It has also been recognized in a few additional cases: two self-incompatible hermaphrodite genetic morphs in *Phillyrea angustifolia* (Oleaceae) were proposed to favor a third morph, which is all-male and fertilizes both hermaphrodite types (Saumitou-Laprade et al., 2010). Similarly, two self-incompatible mating groups occur in *Fraxinus excelsior*, one of which produces most of the fruits. This mating system renders *F. excelsior* functionally subdioecious (Saumitou-Laprade et al., 2018). These findings support a role for within-morph incompatibility in promoting the evolution of dioecy, as suggested by our model. Whether within-morph incompatibility also occurs in natural populations of *Ziziphus* requires further study. When within-morph compatibility is assumed (scenarios 5 and 7 in **Table 2**), the Early morph often goes extinct. The Late morph then diverges into a male-function genotype and female-function genotype, which take advantage of the potential for within-morph crossing.

Within-plant self-incompatibility has been demonstrated in some *Ziziphus* species (Galil and Zeroni, 1967; Asatryan and Tel-Zur, 2013). Nevertheless, our model does not predict a role for such within-genotype incompatibility in selecting for dioecy. Whether within-genotype fertilization was allowed or excluded from the simulations did not affect the trees’ predicted mating system. Within-genotype self-incompatibility may play a different evolutionary role in *Ziziphus*. Protandry and self-incompatibility often occur together in plants but were hypothesized to serve different functions, based on phylogenetic reconstruction models: protandry was proposed to reduce physical interference between the flowers’ male and female functions, while self-incompatibility was suggested to maintain genetic variation by preventing inbreeding (Routley et al., 2004).

An important prediction of our model is that subdioecy can readily evolve from heterodichogamy, suggesting that the co-existence of two morphs within the mating system is unstable. Additional costs of hermaphroditism, such as the need to produce both male and female sexual organs by each individual plant, may push the mating system towards full dioecy. While such costs were not part of the current model, they can be readily added to the framework for future analyses. A full evolutionary transition towards dioecy would require complete sexual specialization into male and female individuals, as well as synchronization in the timing of flower opening in the two sexes. Further studies are needed to elucidate the complex interaction between pollinators’ foraging patterns and the reproductive system of heterodichogamous plants.

## Data Availability Statement

The datasets generated for this study are available on request to the corresponding author.

## Author Contributions

NT-Z, OR-B, SL-Y, and TK planned the experiments. IS, NT-Z, TK, UZ, and YL performed field observations and analyzed the data. EW generated the simulation model. EW and TK wrote the manuscript and all authors commented on it.

## Funding

This work was supported by USAID-MERC, grant no. TA-MOU-12-M32-021.

## Conflict of Interest

The authors declare that the research was conducted in the absence of any commercial or financial relationships that could be construed as a potential conflict of interest.
